# A study of the safety and efficacy of multi-mode NMR-guided double-antiplatelet pretreatment combined with low-dose rtPA in the treatment of acute mild ischemic stroke

**DOI:** 10.3389/fneur.2025.1482078

**Published:** 2025-03-05

**Authors:** Xia Li, Yan Chen, Zhong Dong, Fangfang Zhang, Ping Chen, Peilan Zhang

**Affiliations:** ^1^Clinical College of Neurology, Neurosurgery and Neurorehabilitation, Tianjin Medical University, Tianjin, China; ^2^Department of Neurology, Tianjin Huanhu Hospital, Tianjin, China

**Keywords:** magnetic resonance imaging, mild stroke, ischemic stroke, alteplase, aspirin, clopidogrel, dual antiplatelet, recombinant tissue-type plasminogen activator

## Abstract

**Objective:**

The objective of this study was to evaluate the safety and efficacy of dual antiplatelet pretreatment combined with low-dose rtPA therapy in patients with acute mild ischemic stroke, guided by multimodal MRI.

**Methods:**

In this study, 383 patients with acute mild ischemic stroke (NIHSS ≤ 5) who had symptom onset within 4.5 hours of MRI screening were selected. Patients in the dual antiplatelet pretreatment plus low-dose rtPA group (164 cases) received dual antiplatelet therapy combined with low-dose (0.6 mg/kg) rtPA intravenous thrombolysis. Patients in the standard-dose group (112 cases) received conventional-dose (0.9 mg/kg) rtPA intravenous thrombolysis. Additionally, patients in the dual antiplatelet group who did not receive intravenous thrombolysis (107 cases) underwent 21 days of oral dual antiplatelet treatment.

**Results:**

There was no significant difference in the baseline NIHSS scores among the three groups before treatment (*p* > 0.05). The proportion of early neurological improvement within 24 hours and within 7 days was significantly higher in the DAPT plus low-dose group compared to both the standard-dose group and the DAPT group, with statistical significance (*p* < 0.05). After 90 days of follow-up, the proportion of good functional outcomes in the DAPT plus low-dose group was significantly higher than in both the standard-dose group and the DAPT group (*p* < 0.05), but there was no significant difference between the standard-dose group and the DAPT group. Safety studies indicated that, under MRI guidance, the DAPT plus low-dose group and the standard-dose group had lower incidences of intracranial hemorrhage transformation and symptomatic intracranial hemorrhage, with no statistical difference among the three groups (*p* > 0.05). Mortality rates were also similar across the three groups (*p* > 0.05), with only one patient passing away in the standard-dose group.

**Conclusion:**

After dual antiplatelet pretreatment combined with low-dose rtPA intravenous thrombolysis for acute mild stroke under multimodal MRI guidance, the proportion of patients with good functional outcomes within 90 days was higher compared to the DAPT group and the standard-dose group, with statistical significance. There was no significant increase in the risk of cerebral hemorrhage compared to the standard-dose group and the DAPT group.

## 1 Introduction

Worldwide, mild strokes are quite common. Statistics show that more than 50% of individuals with acute ischemic stroke also experience mild ischemic stroke (1, 2). For acute ischemic stroke within 4.5 h of onset, intravenous thrombolysis with alteplase is the most well-supported pharmacological therapy according to evidence-based medicine. However, there is controversy regarding the use of intravenous thrombolysis for patients with mild strokes (3–6). Most experts agree that acute reperfusion therapy may pose more risks than benefits for those with mild strokes, who generally have a good prognosis. Nevertheless, the prognosis and clinical outcomes for these patients could still be improved. The MaRISS study found that 37% of patients with mild ischemic stroke (NIHSS ≤5) had functional disability at 90 days (mRS score of 2–6) (5). The administration of intravenous thrombolysis, as well as the dose of alteplase (recombinant tissue-type plasminogen activator, rt-PA), affects the prognosis of ischemic stroke patients. The ENCHANTED study (7) showed that low-dose rt-PA (0.6 mg/kg) can reduce mortality and disability after 90 days, but it did not establish that this treatment is superior to standard-dose intravenous thrombolysis. However, the incidence of symptomatic intracerebral hemorrhage (sICH) was lower in the low-dose group. A meta-analysis comparing the effects of standard-dose and low-dose rt-PA intravenous thrombolysis (8) showed that both doses were equally effective. However, the incidence of intracranial hemorrhage increased with the dose of rt-PA (9). Multimodal MRI examinations can effectively reflect the condition of the responsible blood vessels and aid in assessing the risk of disease progression. MRI-guided thrombolytic therapy appears to be both safe and effective for patients with low NIHSS scores or no disability (10).

This study examined the safety and efficacy of dual antiplatelet pretreatment combined with low-dose rtPA intravenous thrombolysis therapy in patients with acute mild ischemic stroke under MRI guidance. The goal was to provide a theoretical basis for using this combined approach in patients with mild ischemic stroke.

## 2 Materials and methods

### 2.1 Research subjects

**Figure 1:**
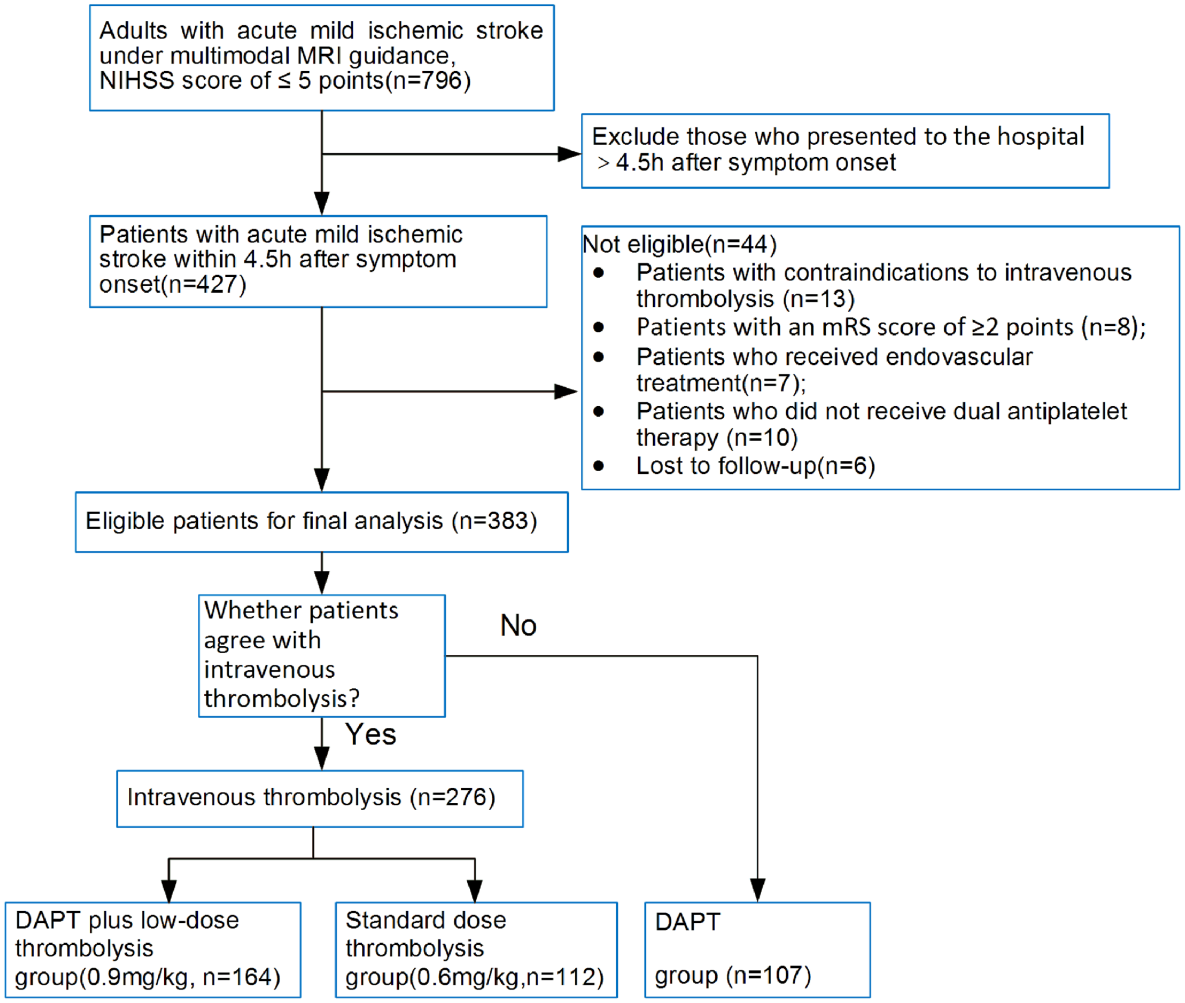
Flowchart of patient selection.

A retrospective study was conducted involving 383 patients with mild stroke who visited Tianjin Huanhu Hospital from May 2021 to March 2024 and completed multimodal MRI examinations (including T1WI, T2WI, DWI, T2-FLAIR, GRE-T2*, and MRA) before treatment (Figure 1). The cohort consisted of 269 males and 114 females, aged 28–85 years. They were divided into three groups based on treatment: the DAPT plus low-dose thrombolysis group (164 cases), which received dual antiplatelet pretreatment combined with rt-PA (0.60 mg/kg) intravenous thrombolysis; the standard-dose thrombolysis group (112 cases), which received rt-PA (0.90 mg/kg) intravenous thrombolysis; and the DAPT group (107 cases), which refused intravenous thrombolysis.

#### 2.1.1 Inclusion criteria

1. Patients met the indications for intravenous thrombolysis as outlined in the “Guidelines for the Diagnosis and Treatment of Acute Ischemic Stroke in China 2018” (11) and the “2019 American Heart Association/American Stroke Association Guidelines for Early Treatment of Acute Ischemic Stroke” (12). 2. NIHSS score ≤ 5 points (0 points for awareness item) at admission. 3. Onset time ≤ 4.5 h. 4. Patients underwent a multimodal MRI examination (including T1WI, T2WI, DWI, T2-FLAIR, GRE-T2*, MRA) before intravenous thrombolysis, and acute ischemic cerebral infarction lesions were confirmed by DWI (with reference to the ADC map).

#### 2.1.2 Exclusion criteria

1. Patients with contraindications to intravenous thrombolysis as specified in the “2018 Guidelines for the Diagnosis and Treatment of Acute Ischemic Stroke in China” and the “2019 American Heart Association/American Stroke Association Guidelines for the Early Treatment of Acute Ischemic Stroke.” 2. Patients with a modified Rankin Scale (mRS) score ≥ 2 points. 3. Patients who received endovascular treatment. 4. Patients who did not receive dual antiplatelet therapy (except for those with contraindications due to bleeding from thrombolytic therapy).

Patients or their relatives were fully informed of the treatment plan and provided signed informed consent (Ethics Committee Approval No.: 2022–047).

### 2.2 Methods

#### 2.2.1 DAPT plus low-dose thrombolysis group

DAPT plus low-dose thrombolysis group: Patients received 100 mg of aspirin orally before thrombolysis, within 4.5 h of onset, combined with 75 mg of clopidogrel pretreatment. They were then administered alteplase rt-PA (Boehringer Ingelheim, Germany, Aileton; specifications: 20 mg and 50 mg, S20110051) at a dosage of 0.60 mg/kg for intravenous thrombolysis (with a maximum dose not exceeding 60 mg). Treatment Method: Fifteen percent of the rt-PA dose was administered intravenously within 1 min, while the remaining 85% was infused intravenously at a constant rate over 60 min. During thrombolysis, the patient’s neurological symptoms and bleeding were closely monitored, and a head CT was reviewed as needed. If the patient’s condition was stable, a multimodal MRI of the head was performed 24 h after thrombolysis. If there was a need to exclude bleeding, a head CT was conducted. Imaging examinations were used to rule out intracranial hemorrhage or bleeding in other critical organs, and the patient was prescribed dual antiplatelet therapy: aspirin 100 mg daily and clopidogrel 75 mg daily for 21 days.

#### 2.2.2 Standard dose thrombolysis group

Standard dose thrombolysis group: Patients were administered alteplase rt-PA (Boehringer Ingelheim, Germany, Alite, specifications: 20 mg and 50 mg, S20110051) within 4.5 h of onset, with a dosage of 0.90 mg/kg for intravenous thrombolysis (maximum dose not exceeding 90 mg). Ten percent of the rt-PA dose was injected intravenously within 1 min, and the remaining 90% was infused at a constant rate over 60 min. During the thrombolysis process, the patient’s neurological symptoms and bleeding were closely monitored, and a head CT was reviewed as necessary. If stable, a multimodal MRI of the head was performed 24 h after thrombolysis. If there was a concern about bleeding, a head CT was conducted. Imaging examinations were used to rule out intracranial hemorrhage or bleeding in other critical organs, and dual antiplatelet therapy was given with aspirin 100 mg daily and clopidogrel 75 mg daily for 21 days.

#### 2.2.3 DAPT group

After completing the multimodal MRI examination, dual antiplatelet therapy was initiated, with aspirin 100 mg daily and clopidogrel 75 mg daily for 21 days.

### 2.3 Evaluation of efficacy

According to the NINDS clinical trial and evaluation criteria (13), NIHSS scores were assessed at admission, 24 h, and 7 days after treatment. Short-term prognosis was considered good if the neurological impairment score improved by ≥ 4 points or if clinical symptoms completely resolved, resulting in an NIHSS score of 0. A poor prognosis was indicated by a decrease in the NIHSS score of less than 4 points at 24 h and 7 days after treatment. Neurological prognosis was further evaluated using the mRS score at 90 days of follow-up, with scores of 0 to 2 indicating a good functional outcome.

### 2.4 Safety evaluation

According to the criteria of the European Collaborative Acute Stroke Study II (ECASS II) (14), symptomatic intracerebral hemorrhage (sICH) is defined as bleeding in any part of the brain identified by CT scan, which directly exacerbates the patient’s clinical symptoms or indicates a poor prognosis, such as increased lethargy, worsening hemiplegia, or an increase in the NIHSS score by more than 4 points.

### 2.5 Statistical analysis

All data were analyzed using IBM SPSS Statistics 29.0. Measurement data that conformed to a normal distribution were expressed as mean ± standard deviation (x̅ ± s), with comparisons between groups performed using the independent sample t-test for two groups or one-way analysis of variance for three or more groups. Data that did not conform to a normal distribution were expressed as the median and interquartile range (M [P25, P75]), with the Mann–Whitney U test and Kruskal-Wallis test applied. Count data were expressed as frequency (*n*) and percentage (%). Comparisons between count data groups were performed using the chi-square test or Fisher’s exact test, depending on the conditions, with *p* < 0.05 considered statistically significant.

## 3 Results

### 3.1 General clinical data of patients with mild ischemic stroke

There were no statistically significant differences in gender, age, hypertension, diabetes mellitus, stroke history, coronary artery disease, atrial fibrillation, smoking status, alcohol use, random blood glucose, systolic blood pressure, diastolic blood pressure, white blood cell count, triglycerides, total cholesterol, low-density lipoprotein, high-density lipoprotein, fibrinogen (FIB), time from onset to medication, proportion of mRS score 0–1 before onset, baseline NIHSS score, proportion of each stroke type in the TOAST classification, proportion of responsible large artery occlusion, and proportion of anterior and posterior circulation among the three patient groups (*p* > 0.05). See Table 1.

**Table 1:** Baseline characteristics of acute mild ischemic stroke patients in the three groups.

Characteristics	DAPT plus low dose group (*n* = 164)	Standard dose group (*n* = 112)	DAPT group (*n* = 107)	*P*
Male Sex [*n* (%)]	118 (72.0)	82 (73.2)	69 (64.5)	0.301
Age (years)	62.3 ± 9.6	60.4 ± 12.1	62.8 ± 9.3	0.115
Hypertension [*n* (%)]	122 (74.4)	72 (64.3)	80 (76.5)	0.138
Diabetes mellitus [*n* (%)]	44 (26.8)	35 (31.3)	36 (33.6)	0.462
Any stroke [*n* (%)]	35 (21.3)	22 (19.6)	31 (29.0)	0.210
Coronary artery disease [*n* (%)]	26 (15.9)	21 (18.8)	14 (13.1)	0.519
Atrial fibrillation [*n* (%)]	8 (4.9)	9 (8.0)	5 (4.7)	0.463
Current smoker [*n* (%)]	60 (36.6)	38 (33.9)	37 (34.6)	0.889
Alcohol use [*n* (%)]	38 (23.5)	21 (18.8)	20 (18.7)	0.530
Baseline blood pressure (mmHg)				
Systolic blood pressure	151.9 ± 19.5	152.1 ± 16.5	153.6 ± 24.0	0.781
Diastolic blood pressure	88.0 ± 11.6	87.5 ± 11.2	87.1 ± 16.3	0.856
Random blood glucose (mmol/L)	7.3 ± 2.4	7.6 ± 2.2	7.1 ± 2.4	0.276
WBC (×10^9^/L)	7.5 ± 2.0	7.9 ± 2.1	7.5 ± 2.2	0.160
Total cholesterol (mmol/L)	5.0 ± 1.1	5.2 ± 1.1	5.0 ± 1.3	0.412
Triglycerides (mmol/L)	1. 6 ± 1.0	1.8 ± 1.3	1.6 ± 1.1	0.399
LDL cholesterol (mmol/L)	3.1 ± 0.9	3.3 ± 0.8	3.2 ± 1.0	0.187
Fibrinogen FIB	2.9 ± 0.9	2.8 ± 0.7	2.9 ± 0.7	0.253
Baseline NIHSS score	3.0 (1.0, 4.0)	3.0 (1.0, 4.0)	3.0 (1.0, 4.0)	0.441
OTT time (minutes)	216.6 ± 35.1	208.1 ± 42.6	213.6 ± 44.2	0.226
mRS score before symptom onset: 0–1 [*n* (%)]				0.218
mRS score 0	156 (95.1)	110 (98.2)	100 (93.5)	
mRS score 1	8 (4.9)	2 (1.8)	7 (6.5)	
TOAST classification [*n* (%)]				0.733
Large artery atherosclerosis	82 (50.0)	46 (41.1)	55 (51.4)	
Small artery occlusion	50 (30.5)	44 (39.3)	31 (29.0)	
Cardioembolism	5 (3)	5 (4.5)	3 (2.8)	
Other determined cause	6 (3.7)	6 (5.4)	5 (4.7)	
Undetermined cause	21 (12.8)	11 (9.8)	13 (12.1)	
Microbleeds ≤ 10 [*n* (%)]	5 (3.0)	6 (5.4)	4 (3.7)	0.620
Intracranial aneurysm <10 mm [*n* (%)]	18 (11.1)	7 (6.3)	5 (4.7)	0.119
Circulation classification [*n* (%)]				0.994
Anterior circulation	97 (59.15)	64 (57.14)	63 (58.88)	
Posterior circulation	65 (39.63)	47 (41.96)	43 (40.19)	
Anterior and posterior circulation	2 (1.22)	1 (0.89)	1 (0.93)	
Occlusion of the responsible large artery [*n* (%)]	34 (20.7)	32 (28.6)	21 (19.6)	0.208

Data are presented as *n* (%), mean ± standard deviation, or *M* (P25,P75), and *p* values were compared among the three groups.

### 3.2 Efficacy and safety analysis

Patients with acute mild ischemic stroke were treated using three methods: the DAPT low-dose group received dual antiplatelet pretreatment combined with (0.6 mg/kg) rtPA intravenous thrombolysis; the standard-dose group received (0.9 mg/kg) rtPA intravenous; and the DAPT group received aspirin and clopidogrel dual antiplatelet therapy. Short-term prognosis was assessed by comparing early neurological improvement within 24 h and 7 days after treatment, while long-term neurological function was evaluated based on good functional outcomes at a 90-day follow-up (see Table 2).

**Table 2:** Comparison of therapeutic effectiveness among the three groups [*n* (%)].

Outcome	DAPT plus low dose group (*n* = 164)	Standard dose group (*n* = 112)	DAPT group (*n* = 107)	*P*
Early neurological improvement within 24 h	117 (71.3)^a1^	44 (39.3)^b1^	25 (23.4)^c1^	<0.01*
Early neurological improvement within 7 d	142 (86.6)^a2^	61 (54.5)^b2^	46 (43.0)^c2^	<0.01*
mRS score 0–2 within 90 d	153 (93.3)^a3^	93 (83.0)^b3^	90 (84.1)^c3^	0.016*

Data are presented as *n* (%), and *p* values were compared among the three groups. * indicates *p* < 0.05, indicating that the difference was statistically significant. ^a1^: Comparison of neurological improvement within 24 h between the DAPT plus low-dose group and the standard-dose group; ^b1^: Comparison between the standard-dose group and the DAPT group within 24 h; ^c1^: Comparison between the DAPT plus low-dose group and the DAPT group within 24 h. ^a2^: Comparison of neurological improvement between the DAPT plus low-dose group and the standard-dose group within 7 d; ^b2^: Comparison between the standard-dose group and the DAPT group within 7 d; ^c2^: Comparison between the low-dose group and the DAPT group within 7 d; ^a3^: Comparison of the proportion of good functional outcomes between the DAPT plus low-dose group and the standard-dose group within 90-d mRS score 0–2; ^b3^: Comparison between the standard-dose group and the DAPT group within 90 days; ^c3^: Comparison between the DAPT plus low-dose group and the DAPT group within 90 d.

Recent neurological function evaluations showed that NIHSS scores at 24 h and 7 days post-treatment were lower than those before treatment (Table 3). For early neurological improvement within 24 h, the difference between the DAPT plus low-dose group and the standard-dose group was significant (^a1^P < 0.05). Significant differences were also observed between the standard-dose group and the DAPT group (^b1^P < 0.05), and between the DAPT plus low-dose group and the DAPT group (^c1^P < 0.05). For early neurological improvement at 7 days, the difference between the DAPT plus low-dose group and the standard-dose group was significant (^a2^P < 0.05). Significant differences were found between the DAPT plus low-dose group and the DAPT group (^c2^P < 0.05), while the difference between the standard-dose group and the DAPT group was not statistically significant (^b2^P > 0.05).

**Table 3:** Comparison of baseline NIHSS scores and 24 h, 7-d NIHSS scores in the three groups.

NIHSS score	DAPT plus low dose group (*n* = 164)	Standard dose group (*n* = 112)	DAPT group (*n* = 107)	*p*
Baseline NIHSS score	3 (1–4)	3 (1–4)	3 (1–4)	0.441
24-h NIHSS score	0 (0–1)	2 (0–4)	2 (1–4)	<0.001
7-d NIHSS score	0 (0–0)	1 (0–3)	1 (0–4)	<0.001

**Figure 2:**
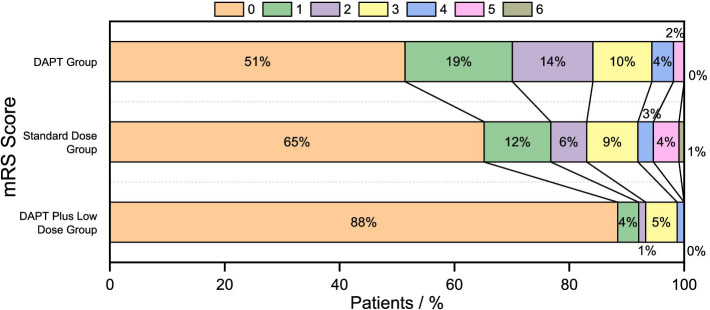
90-days mRS score displacement.

In terms of long-term neurological prognosis, the results from the 90-day follow-up of the three groups showed the following proportions of good functional outcomes: 153 cases (93.3%) in the DAPT plus low-dose group, 93 cases (83.0%) in the standard-dose group, and 90 cases (84.1%) in the DAPT group. Compared to the standard-dose and DAPT groups, the DAPT plus low-dose group showed a statistically significant difference (^a3,c3^P < 0.05), while the difference between the standard-dose group and the DAPT group was not statistically significant (^b3^P > 0.05). The good functional outcomes in the DAPT plus low-dose group were more pronounced, with significant differences among the three groups (*p* = 0.016). Results are shown in Table 2, and the distribution of 90-day mRS scores is illustrated in Figure 2.

Safety studies revealed that the incidence of bleeding, including intracranial hemorrhage transformation and symptomatic intracranial hemorrhage during intravenous thrombolysis under MRI guidance, was lower in the low-dose and standard-dose groups compared to the DAPT group. However, no significant differences among the three groups were observed (*p* > 0.05). Except for bleeding, there were no serious adverse events related to thrombolysis (such as severe laryngeal edema, allergic reactions, liver and kidney function damage, cytopenias, etc.) in the Standard dose group, the DAPT group, the DAPT plus low-dose group. Regarding mortality, only one patient died in the standard-dose group, and there were no significant differences among the three groups (*p* > 0.05). See Table 4.

**Table 4:** Comparison of treatment safety among the three groups [*n* (%)].

Safety indicators	DAPT plus low dose group (*n* = 164)	Standard dose group (*n* = 112)	DAPT group (*n* = 107)	*P*
Intracranial hemorrhage	2 (1.2)	3 (2.7)	0 (0)	0.216
sICH	0 (0)	1 (0.9)	0 (0)	0.314
Non-sICH	2 (1.2)	2 (1.8)	0 (0)	0.648
Death	0 (0)	1 (0.9)	0 (0)	0.302

Data are presented as *n* (%) and *p* values were compared among the three groups.

## 4 Discussion

Current guidelines recommend administering alteplase intravenously to patients with acute ischemic stroke (AIS) who seek medical attention within 4.5 h of symptom onset (11, 12). However, there is controversy regarding intravenous thrombolytic therapy for mild cases (3–5). The “Guidelines for the Diagnosis and Treatment of Acute Ischemic Stroke in China 2018” states (11): For mild non-disabling strokes and strokes with rapid symptom improvement, intravenous thrombolytic therapy can be considered, provided a full evaluation and communication are conducted. The “2019 American Heart Association/American Stroke Association Guidelines for Early Treatment of Acute Ischemic Stroke” (12) recommends intravenous thrombolytic therapy for disabling mild ischemic strokes (NIHSS score 0–5) but not for non-disabling strokes. The definition of disability generally refers to the impact on the patient’s daily life and work, though standards may vary among different patients.

Analysis of a subgroup of patients with mild ischemic stroke in the IST-3 randomized trial demonstrated that intravenous alteplase was superior to standard medical treatment (15). An observational study (16) including 1,386 patients with NIHSS scores of ≤5 found that 194 (14.0%) received intravenous thrombolysis therapy. Results indicated that the thrombolysis group was more effective than the control group, with a statistically nonsignificant risk of symptomatic hemorrhagic transformation.

Multiple international meta-analyses have shown that intravenous thrombolysis is safe and effective in patients who are taking antiplatelet agents in the early stages (17, 18).

Because the half-life of rt-PA in plasma is very short, it is quickly eliminated from the blood after intravenous injection, and only 10% is left after 20 min of medication. With the gradual dissolution of thrombosis, the rupture of unstable plaque and the damaged intima can be exposed again. Thereby inducing and promoting local platelet activation and aggregation. It is easier to form thrombosis in the short term, and even some patients will have arterial re-occlusion, and the symptoms are repeated or aggravated.

Aspirin and Clopidogrel are commonly used antiplatelet aggregation drugs in clinical practice, and their mechanisms of action are different: Aspirin effectively inhibits thromboxane A2 production by acting on cyclooxygenase, thereby inhibiting platelet aggregation (19). While Clopidogrel inhibits platelet aggregation by selectively inhibiting adenosine diphosphate (20). Early antiplatelet therapy after intravenous thrombolysis can reduce early neurological deterioration by preventing vascular re-occlusion and stroke progression (21). In a single-center clinical study from the Netherlands, antiplatelet agents prior to intravenous thrombolysis were shown to be independently associated with a favorable outcome (22).

Recently, meta-analyses of the efficacy of antiplatelet conditioning prior to intravenous thrombolysis have shown that antiplatelet conditioning prior to intravenous thrombolysis reduces the risk of long-term neurological deterioration (18). Low-dose Alteplase has also been shown to improve outcomes in patients with acute ischemic stroke who have received prior antiplatelet therapy with thrombolysis (23).

The study also showed that the proportion of patients with good functional outcomes at 90 days of follow-up was higher in the DAPT plus low-dose group than in both the standard-dose group and the DAPT group, with significant differences (*p* < 0.05). No significant difference was found between the standard-dose group and the DAPT group (*p* > 0.05). These findings suggest that dual antiplatelet pretreatment combined with low-dose rtPA intravenous thrombolysis is more effective for treating acute mild ischemic stroke than traditional dual antiplatelet therapy and standard-dose rtPA thrombolysis. Additionally, the proportion of patients showing early neurological improvement within 7 days was significantly higher in the DAPT plus low-dose group compared to the standard-dose group and the DAPT group (*p* < 0.05), while the difference between the standard-dose group and the DAPT group was not significant (*p* > 0.05). This suggests that dual antiplatelet pretreatment combined with low-dose rtPA therapy provides a faster onset of effect and significantly improves early neurological function.

The SITS registry reported that 25% of mild strokes were complicated by intracranial and extracranial large vessel occlusions, consistent with the findings of this paper (24). Studies have indicated that intravenous thrombolytic therapy is an independent predictor of good functional prognosis at 90 days for patients with mild non-disabling stroke and severe large vessel stenosis/occlusion (25). Several studies have also shown that patients with acute ischemic mild stroke and large artery occlusion are at high risk of progressive neurological deterioration, particularly those with posterior circulation cerebral infarction, who are critically ill and progress rapidly. Conventional doses of antiplatelet therapy, cerebral circulation improvement, and statin therapy for atherosclerosis are not optimal. Arterial thrombolysis is limited by technology, equipment, and its time-consuming nature, making it difficult to implement widely. Intravascular stent therapy for the posterior circulation system is also risky and challenging. Studies from the German Stroke Registry-Endovascular Treatment (GSR-ET) and the Safe Implementation of Treatments in Stroke-International Stroke Thrombolysis Register (SITS-ISTR) databases indicate that for patients with acute mild stroke (NIHSS score ≤ 5) and large vessel occlusion, intravenous thrombolysis results in a 2.16-fold improvement in 90-day functional outcomes compared to bridging therapy or direct mechanical thrombectomy (26). Antiplatelet therapy has been shown to be effective in acute ischemic stroke with macrovascular disease in recent studies (27, 28).

All patients in this study underwent multimodal MRI examinations before treatment. Multimodal MRI can detect ischemic lesions within minutes of symptom onset, providing early information on lesion size, location, and timing. It is more sensitive than conventional MRI for detecting small infarct lesions. Magnetic Resonance Angiography (MRA) can reveal occlusion or stenosis of large blood vessels in the head. Multimodal MRI and MRA examinations provide a clear view of the condition of the responsible blood vessels and support early treatment decisions for patients with mild ischemic stroke (17). This safety study found that, compared to the DAPT group, both the DAPT plus low-dose group and the standard-dose group under MRI guidance had lower rates of intracranial hemorrhage transformation and symptomatic intracranial hemorrhage, with no significant statistical difference among the three groups (*p* > 0.05). Mortality rates were not statistically different among the groups (*p* > 0.05), with only one patient passing away in the standard-dose group. The study concludes that intravenous thrombolysis guided by multimodal MRI can be beneficial and safe for patients with cerebral infarction, large vessel occlusion, and posterior circulation infarction.

In summary, for patients with mild stroke, opting for dual antiplatelet pretreatment guided by multimodal MRI and combined with low-dose rt-PA intravenous thrombolysis may reduce the risk of bleeding associated with intravenous thrombolysis and enhance patient prognosis. Future multicenter, long-term studies with large sample sizes are needed to further validate these findings and provide more individualized treatment options for acute mild ischemic stroke (MIS) patients.

## Data Availability

The original contributions presented in the study are included in the article/supplementary material, further inquiries can be directed to the corresponding author.
